# Monoclonal antibodies to 65kDa glutamate decarboxylase induce epitope specific effects on motor and cognitive functions in rats

**DOI:** 10.1186/1750-1172-8-82

**Published:** 2013-06-05

**Authors:** Christiane S Hampe, Laura Petrosini, Paola De Bartolo, Paola Caporali, Debora Cutuli, Daniela Laricchiuta, Francesca Foti, Jared R Radtke, Veronika Vidova, Jérôme Honnorat, Mario Manto

**Affiliations:** 1 University of Washington, School of Medicine, SLU S-276, Seattle, WA, 98109, USA; 2 IRCCS Santa Lucia Foundation, Rome, Italy, Dept of Psychology, University “Sapienza” of Rome, Rome, Italy; 3 IRCCS Santa Lucia Foundation, Rome, Italy, Dept of Developmental and Social Psychology, University “Sapienza” of Rome, Rome, Italy; 4 Centre de Référence, de Diagnostic et de Traitement des Syndromes Neurologiques Paranéoplastiques, Hospices Civils de Lyon, Université de Lyon, Claude Bernard Lyon 1, Lyon Neuroscience Research Center INSERM U1028/CNRS UMR 5292, Lyon, France; 5 Unité d’Etude du Mouvement, FNRS Neurologie, ULB Erasme, Brussels, Belgium

**Keywords:** Glutamate decarboxylase, Monoclonal antibodies, Morris Water Maze, Neurological, Evaluation

## Abstract

**Background:**

Stiff Person Syndrome (SPS) is a rare autoimmune movement disorder characterized by the presence of autoantibodies specific to the smaller isoform of glutamate decarboxylase (GAD65). A pathological role of these antibodies has been suggested by their capacity to inhibit GAD65 enzyme activity and by the observation that rats receiving cerebellar injections of GAD65Ab showed cerebellar motor hyperexcitability. To assess the effect of epitope-specific GAD65Ab on cognitive and motor functions, we conducted behavioral experiments in rats that received cerebellar injections with two distinct monoclonal GAD65Ab (b96.11 and b78).

**Methods:**

Rats received three injections of GAD65Ab b96.11 (5 or 7 μg), GAD65Ab b78 (5 or 7 μg), or saline at the level of three cerebellar nuclei. Animals were submitted to neurological evaluation and Morris Water Maze (MWM) test. Cellular internalization of GAD65Ab was analyzed by Flow Cytometry, Fluorescence and Bright Field microscopy.

**Results:**

Monoclonal GAD65Ab induced dose-dependent and epitope-specific effects on motor and cognitive functions. Injections of the higher dose altered motor and spatial procedural behaviors, while the lower dose induced only modest cerebellar motor symptoms and did not affect MWM performances. While b96.11 provoked immediate severe effects, which rapidly decreased, b78 induced moderate but prolonged effects. Both GAD65Ab were taken up by live cells in a dose-dependent manner.

**Conclusions:**

Our findings support the hypothesis that epitope-specific GAD65Ab induce cerebellar dysfunction impairing motor and procedural abilities. This is the first demonstration of a critical role of cerebellar nuclei GAD65 enzyme in procedural spatial functions.

## Introduction

Gamma-Aminobutyric acid (GABA) is the major inhibitory neurotransmitter in the mammalian central nervous system (CNS). Its synthesis from glutamate is catalyzed by two isoforms of glutamate decarboxylase, namely GAD67 and GAD65 [1]. Only GAD65 is recognized by autoantibodies present in patients with Type 1 diabetes (T1D) and autoimmune movement disorders, including Stiff Person Syndrome (SPS) [2] and certain subtypes of Cerebellar Ataxia (CA) [3-5]. GAD65Ab in these three disorders show distinct differences in their tissue distribution and epitope specificities [6]. GAD65Ab in T1D patients are found only in the periphery, while the antibodies are present both in the periphery and the CNS in SPS and CA patients [4,7]. Moreover, only GAD65Ab present in SPS patients inhibit GAD65 enzyme activity [8]. This latter observation together with the finding that SPS patients have reduced levels of GABA in cerebrospinal fluid and brain [9,10] has led to the hypothesis that GAD65Ab may have a pathogenic role. Yet the intracellular location of GAD65 and the assumed impermeability of intact neurons appeared to prevent access of GAD65Ab to their target. However, a recent study by Hill *et al.* challenged this view when they demonstrated that IgG can be directly internalized by Purkinje cells providing access to intracellular antigens [11]. Indeed, it was reported that GAD65Ab down-regulated GABA synthesis in basket cell terminals and thereby reduced GABA release on Purkinje cells, thus selectively suppressing GABAergic neurotransmission [12,13]. We investigated the effect of GAD65Ab on neuronal activity by injecting rat cerebella with IgG purified from GAD65Ab-positive SPS and CA patients [14]. The antibodies impaired N-methyl-D-aspartate (NMDA)-mediated turnover of glutamate, enhanced spinal cord excitability, and altered adaptation of motor cortex to repetitive peripheral stimulation [14]. We concluded that GAD65Ab interfered with the regulation of brain neurotransmitters and thereby impaired neuronal activity. This conclusion was recently confirmed in studies demonstrating that purified IgG from a SPS patient with high GAD65Ab titers induced motor dysfunction in rats [15]. Both SPS and CA are characterized by dysfunctional GABAergic neurotransmission [3,13] but show distinct disease-specific symptoms. CA patients show moderate to severe gait ataxia with mild limb ataxia and nystagmus, while SPS patients show progressive muscular rigidity, predominantly of the trunk muscles, with superimposed spasms [16]. To investigate whether disease-specific GAD65Ab are involved in these clinical distinct phenotypes, we utilized two monoclonal GAD65Ab with well-characterized epitope specificities. One of these antibodies inhibited GAD65 enzyme activity and shared an epitope recognized by GAD65Ab present in SPS patients [17]. Our hypothesis that different GAD65Ab induce distinct neurological effects was verified when we injected rat cerebellar nuclei with these monoclonal GAD65Ab.

Specifically, we found that the SPS-associated monoclonal GAD65Ab induced greater effects regarding increased glutamate concentration, decreased cerebello-cortical inhibition, and impaired corticomotor response [18]. These results could explain the different neurological syndromes observed in GAD65Ab-positive patients.

Based on these results, we now analyzed the effects of intra-nuclear cerebellar injections with two monoclonal GAD65Ab (b96.11 and b78) on postural and spatial behavior in rats. To assess GAD65Ab-induced cerebellar impairment, we submitted treated rats to a detailed neurological evaluation and analyzed their performance in the Morris Water Maze (MWM) test. This testing is widely used to study motor and cognitive function in the evaluation of cerebellar dysfunction [19-23].

## Methods

### Monoclonal antibodies b96.11 and b78

Human monoclonal antibodies b96.11 and b78 specific to GAD65 were derived from a patient with Autoimmune Polyendocrine Syndrome Type 1 [24]. This patient was non-diabetic and showed no symptoms of SPS. Monoclonal antibodies were isolated from supernatants of the respective B cell lines. The protein concentration was adjusted to 1 mg/ml. Both antibodies show binding to human, mouse and rat GAD65 without any reactivity to GAD67. The conformational epitope recognized by b96.11 is also bound by GAD65Ab in ~80% of T1D patients [25] and only by 50% of SPS patients [17]. Remarkably, approximately 30% of SPS patients also present T1D. The conformational epitope recognized by b78 is bound by GAD65Ab present in 10% of T1D patients and 70% of SPS patients [17]. Notably, only b78 inhibits the enzyme activity of GAD65 [17].

### Experimental design

Adult male Wistar rats (N=5/group) were injected in the three deep cerebellar nuclei with 5 or 7 μg of monoclonal GAD65Ab b96.11 (Group names: 5-b96.11, 7-b96.11), GAD65Ab b78 (Group names: 5-b78, 7-b78) or with the respective volumes of saline (5 and 7 μl) (Group names: 5-sham, 7-sham). The doses used are based on previous experiments demonstrating an effect on cerebellar nuclei [14]. All animals were pair- or three-housed and kept under standard conditions with food and water *ad libitum* on a 12/12hrs dark/light cycle. The neurological evaluation and MWM testing started 20 hrs after the injections and lasted four post-injection days.

### Surgery

**Figure 1:**
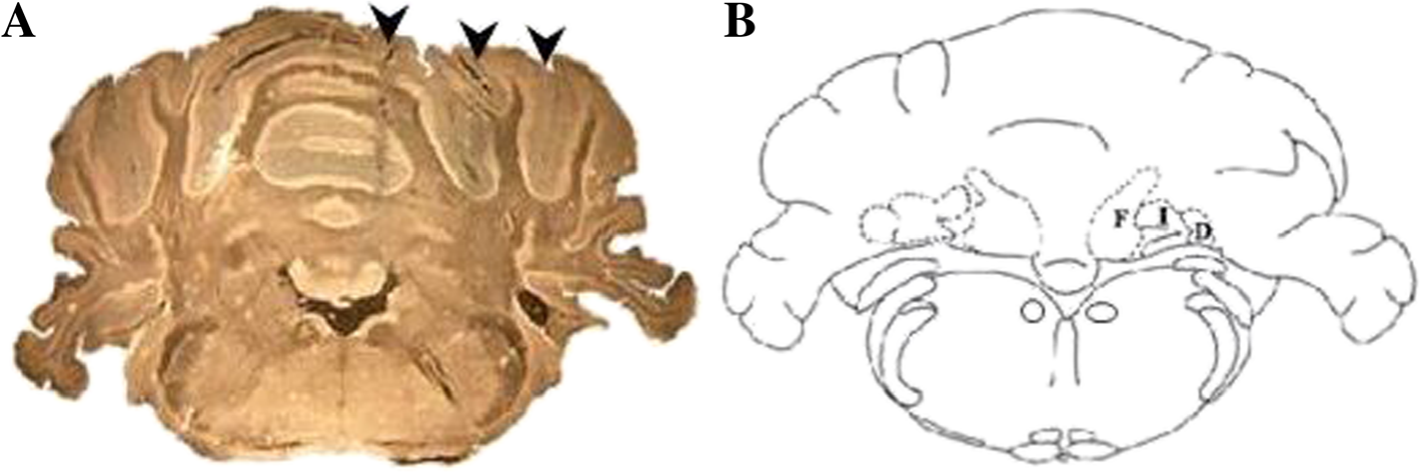
**Illustration of the three unilateral injections in the deep cerebellar nuclei of the right side.** The microphotograph (**A**) shows the three tracks (arrowheads) of the injections into the cerebellar nuclei. The schematic drawing (**B**) of the corresponding coronal section (AP level: between - 11. 6 and -11.3 mm from bregma) shows the three cerebellar nuclei. F: Fastigial; I: Interposed; D: Dentate nucleus.

All rats were anesthetized with Zoletil 100 (Tiletamine and Zolazepam: 50 mg/kg i.p.-Virbac s.r.l., Milan, Italy) and Rompun (Xylazine: 10 mg/Kg i.p.- Bayer s.p.a., Milan, Italy). Three injections were performed at the level of the three right cerebellar deep nuclei (stereotaxic coordinates: fastigial: AP -11.6, ML -1, DV -6.2; interposed: AP -11.3, ML -2.2, DV -6.1; dentate: AP -11.3, ML -3.4, DV -6.1) [26] (Figure 1). Solutions were injected in each nucleus at flow rates of 1 μl/min. The needle was left *in situ* 5 min after each injection. The animals were allowed to recover from anesthesia and surgical stress for 20 hrs.

### Neurological evaluation

The presence of postural symptoms, locomotor handicaps, and complex behavior deficits was assessed by means of a behavioral rating scale [20] (see Additional file 1). A score from 0 to 2 was assigned to each symptom according to its degree of severity (0=absent; 1=slight; 2=marked). As 20 behaviors were taken into

 account, the total score ranged from 0 (complete absence of any deficits) to 40 (presence of all symptoms to the highest degree). The behavioral scores were attributed by an expert investigator unaware of the individual specimen’s group (b96.11, b78 or sham). Behavioral evaluations were repeated for four post-injection days.

### Morris Water Maze (MWM)

To test mnesic and procedural spatial functions in the presence of intra-cerebellarly injected GAD65Ab, we submitted the animals to the MWM test apt to evaluate mnesic (localizatory competencies) and procedural (navigational strategies) performances in animals with cerebellar alterations [19-23]. To evaluate short-lasting GAD65Ab effects we chose an MWM protocol detailing both early (first 48 h) and more delayed effects in analogy with an MWM protocol previously used [21]. The rats were placed in a circular white pool (diameter 140 cm) filled with 24°C water made opaque by the addition of atoxic acrylic black color (Giotto, Italy). An escape platform (diameter 10 cm) was placed in the middle of one quadrant (NW, NE, SW, SE), 30 cm from the side walls. It was either submerged 2 cm or raised 2 cm above the water level. On day 1, each rat was submitted to 10-trial Place 1 phase (hidden platform put in the SE quadrant), followed by 1 trial with no platform in the pool (Probe 1) and then by a 4-trial Cue phase (visible platform in the NW quadrant). Four hours later, the animals performed a 10-trial Place 2 phase (hidden platform in the NW quadrant) followed by Probe 2. On day 2 and 3, the animals performed 10-trial Place 3 (hidden platform in the NE quadrant) and 10-trial Place 4 (hidden platform in the SW quadrant) followed by Probe 3 and Probe 4, respectively.

The rat was released into the water from randomly varied starting points and allowed to search for the hidden or visible platform for a maximum of 120 sec. When the rat reached the platform, it was allowed to remain there for 30 sec. The inter-trial interval was 1 min. In the Probe phases the platform was removed and rats were allowed 60 sec to search for it. Navigational trajectories were recorded by a video camera whose signal was relayed to a monitor and to an image analyzer (Ethovision, Noldus, Wageningen, The Netherlands).

The following MWM parameters were considered: escape latency (in sec) to find the platform; total distance (in cm) swum in the pool; peripheral distance, considered as percentage of total distance swum in a 20-cm peripheral annulus; mean swimming velocity (in cm/sec); percentage of time spent in the previously rewarded (platform) quadrant (× = time in sec/60 sec × 100) during the Probe phase; navigational strategies put into action in reaching the platform. The navigational strategies were classified in six main categories, regardless of whether the platform was reached or not: Circling (C): circular swimming with inversion of direction and counterclockwise and clockwise turnings; Extended Searching (ES): swimming around the pool in all quadrants, visiting the same area more than once; Restricted Searching (RS): swimming in some pool quadrants, not visiting other areas; Loop Searching (LS): swimming around the pool with compulsive counterclockwise or clockwise restricted turnings; Indirect Finding (IF): reaching the platform by swimming through a semicircular trajectory; direct Finding (F): swimming towards the platform without any foraging around the pool. Animals were not trained in MWM prior to injections to avoid prior acquisition of skills. Two researchers unaware of the individual specimen’s group categorized the swimming trajectories drawn by the image analyzer. They attributed the dominant behavior in each trial to a specific category. Categorization was considered reliable only when their judgments were consistent.

### Uptake of antibodies by AF5 cells

The AF5 cell line (a kind gift from Dr. William J. Freed, NIH) is an immortalized rat CNS cell line, which differentiates to a neuronal phenotype with GAD65 expression when allowed to grow to full confluency [26-28]. The cells were maintained under permissive conditions in a 5% CO_2_ incubator in Neurobasal Media (Gibco Life Technologies, Gaithersburg, MD) containing B-27 supplement (Gibco Life Technologies), 0.5 mM L-glutamine, 100 U/ml penicillin and 100 μg/ml streptomycin.

Expression of intracellular GAD65 in AF5 cells was established using monoclonal GAD65 specific antibody N-GAD65mAb (mouse) [29]. AF5 cells (1x10^6^) were fixed with 4% paraformaldehyde in phosphate-buffered saline (PBS) for 7–10 min and then permeabilized with ethanol: acetic acid (95:5) for 1–2 min at 20°C. N-GAD65mAb was applied to the cells for 1 hour at 4°C. After washes, bound N-GAD65mAb was detected by secondary Tetramethyl Rhodamine Isothiocyanate (TRITC)- labeled anti-mouse antibody (AbD Serotec, Raleigh, NC). Cells were washed twice and examined by flow cytometry (FACScan; Becton Dickinson, San Jose, CA).

### Internalization of GAD65Ab analyzed by flow cytometry

Monoclonal GAD65Ab b96.11 and b78 were labeled with Alexa Fluor 488 according to the manufacturer’s instructions (Invitrogen, Carlsbad, CA). AF5 cells (1×10^6^/100 μl) were incubated with b96.11-Alexa-488 or b78-Alexa-488 (both at 1.6 μM) for the indicated times at 37°C. After washes, cells were analyzed by flow cytometry as above.

### Internalization of GAD65Ab analyzed by fluorescence and bright field microscopy

AF5 (1×10^6^/100 μl) cells were incubated with b78-Alexa-488, b96.11- Alexa-488, or HAA1-Alexa 647 (all at 1.6 μM) for 4 hours at 37°C. After the last wash step, cells were stained with SYTOX-Orange (Invitrogen) according to the manufacturer’s instructions. Cells were placed on a 35-mm Petri dish with glass bottom (cover glass No. 1.5, MatTek, MA, USA), covered with Slow Fade Light mounting solution or buffer (Molecular Probes, Eugene, OR) and immediately analyzed. Images were obtained with objective PlanApoN 60×/1.42 NA, oil (Olympus) with image size 1024×1024 pixels. Cells were visualized in bright field fluorescence mode using the appropriate filter (FITC for Ex/Em 490/525 nm (Alexa 488), Cy5 for Ex/Em 645/705 (Alexa 647), TRITC for Ex/Em 555/605 (SYTOX-Orange)) and images were processed by the SoftWorX program (Version 5.0.0, Applied Precision, WA, USA). Intensity scale of the respective channel was adjusted manually in the histogram of Scale Image dialog box to remove fluorescence background of control cells (AF5 cells incubated without labeled antibody). The images reported in the figures are representative of at least three experiments that gave similar results.

### Statistical analysis

Data were presented as mean ± SEM and were tested for normality (Will-Shapiro’s test) and homoscedasticity (Levene’s test). When data distribution was not normal, as for neurological evaluation scores, we applied the non-parametric Mann-Whitney U test to compare total, postural and locomotor symptoms and complex behaviors. MWM data were analyzed using one-way ANOVAs (group) or two-way ANOVAs for independent (group) and repeated measures (strategies) followed by Tukey’s test, when appropriate. To control for the alpha inflation the proportion of type I errors among all rejected null hypotheses the False Discovery Rate (FDR) was set to 0.05. The FDR was estimated through the procedure described by Storey and Tibshirani [30]. The bootstrap procedure was used to estimate the π0 parameter [31]. In our results, the 0.05 level of significance corresponded to an FDR 0.03.

## Results

### Animals injected with 7 μg of GAD65Ab

#### Neurological evaluation

**Figure 2:**
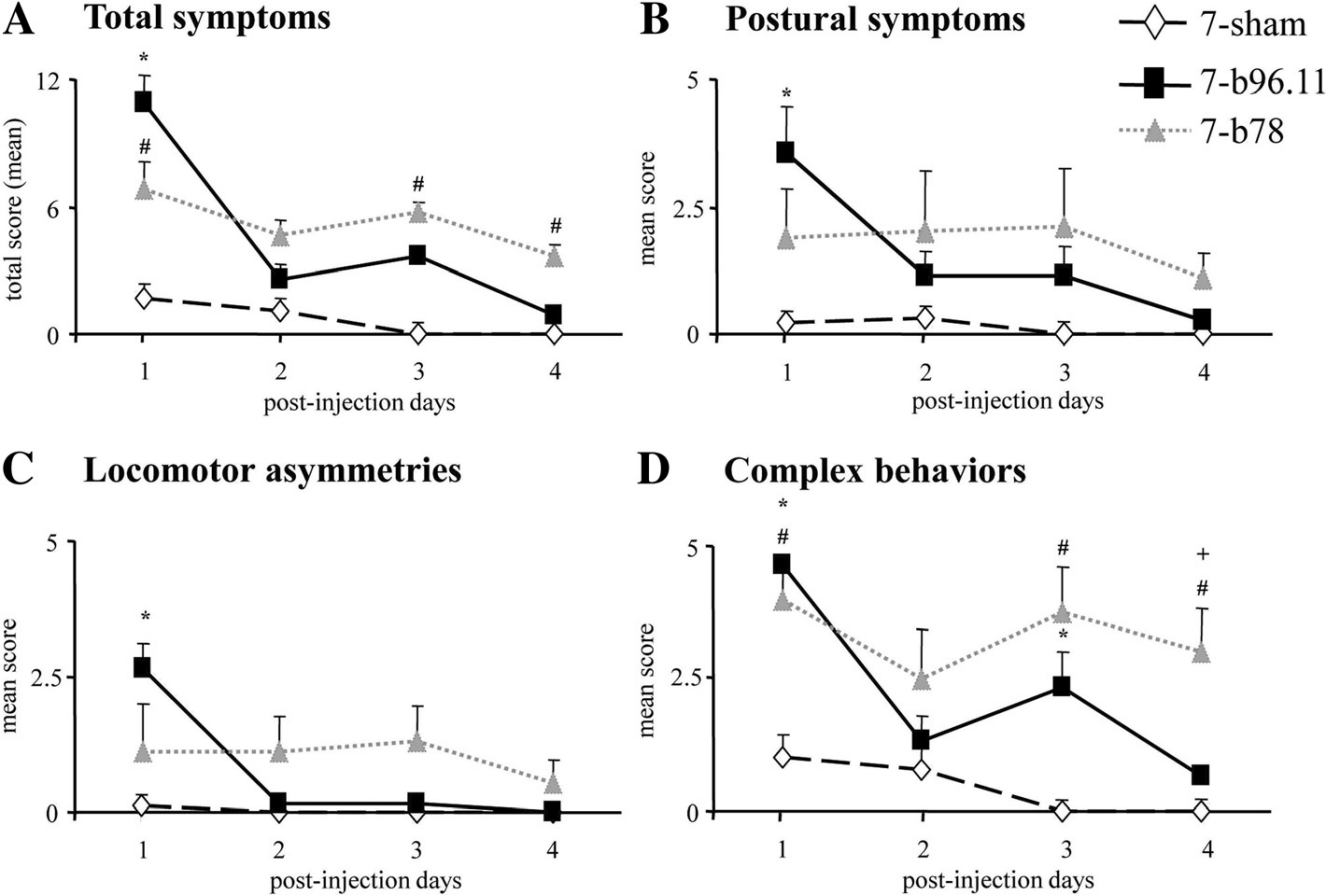
**Neurological evaluation of total symptoms (A), postural symptoms (B), locomotor asymmetries (C) and complex behaviors (D) in 7-b96.11 (black squares), 7-b78 (grey triangles), and 7-sham (white diamonds) groups.** (7-b96.11 *vs.* 7-sham: * *p* <0.03; 7-b78 *vs.* 7-sham: # *p* <0.03; 7-b96.11*vs.* 7-b78: **+***p* <0.03).

Twenty hours after injections, 7-b96.11 animals showed more severely impaired motor behaviors compared to 7-sham animals. However, the motor impairment decreased after the first day so that the 7-b96.11 rats no longer differed from 7-sham animals (Figure 2A). In contrast, the 7-b78 rats exhibited slight motor impairment (as compared to 7-sham rats) lasting for the entire evaluation period (Figure 2A). Both GAD65Ab-treated groups exhibited motor symptoms resembling those of a right cerebellar dysfunction. To further characterize motor behavior, the behavioral rating scale was sub-divided in postural symptoms, locomotor asymmetries and complex behaviors. When analyzing the postural symptoms alone, we found that 7-b96.11 rats were impaired on post-injection day 1, after which the symptoms rapidly decreased (Figure 2B). The same pattern was observed for locomotor symptoms, where the impairment displayed by 7-b96.11 rats was more severe compared to 7-sham rats on post-injection day 1, after which the symptoms disappeared (Figure 2C). As for complex behaviors, both GAD65Ab-treated groups showed an impairment more severe than observed in the 7-sham group. On post-injection day 4, only rats in the 7-b78 group were still impaired in comparison to 7-b96.11 and 7-sham groups (Figure 2D). Results with significance differences established by the non-parametric Mann-Whitney U test on motor assessment data are depicted in Figure 2.

#### Morris Water Maze

**Figure 3:**
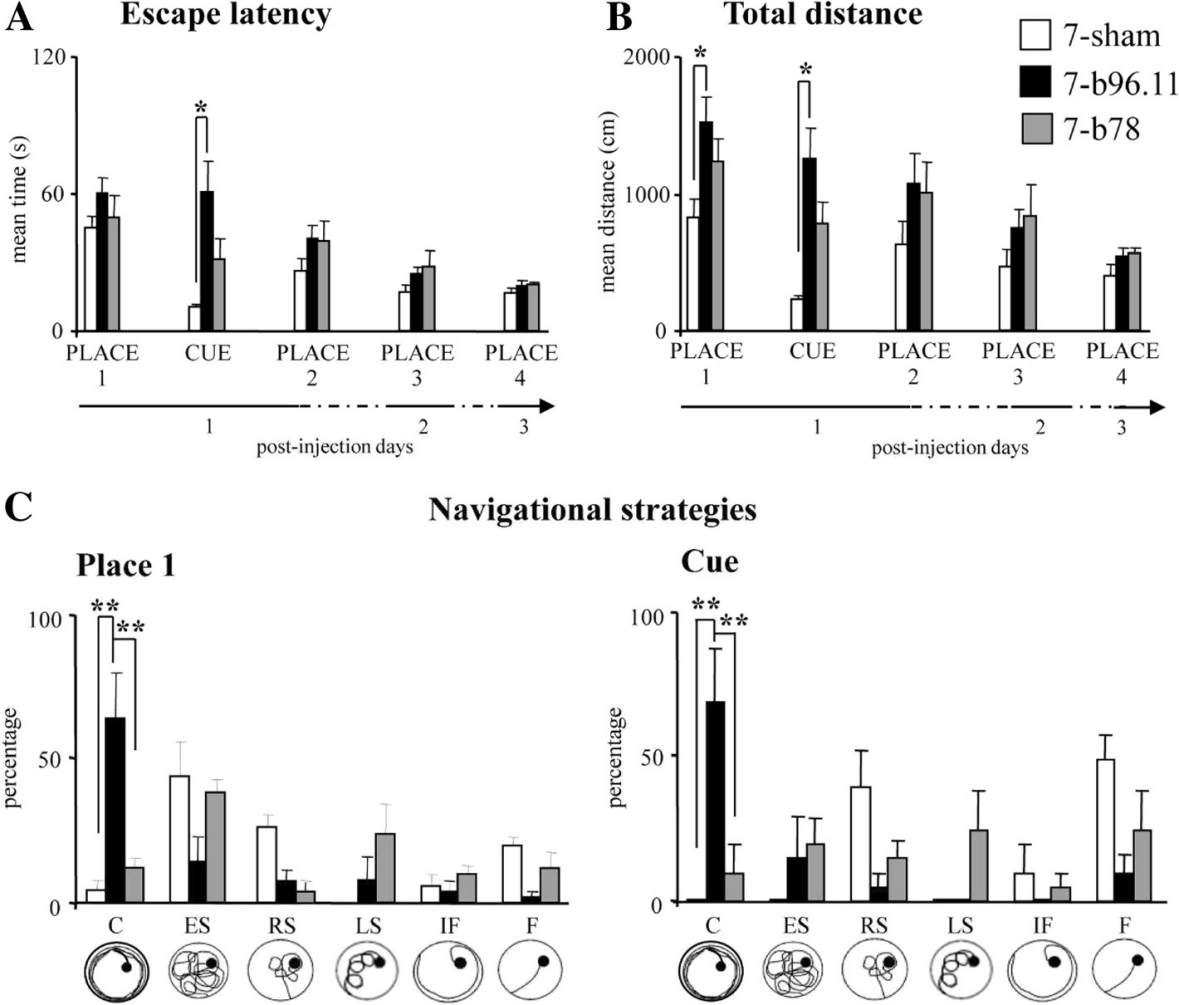
**Comparisons among 7-b96.11 (black columns), 7-b78 (grey columns), and 7-sham (white columns) groups throughout MWM testing.** Escape latency (**A**), Total distance (**B**) and Navigational strategies (**C**) were depicted. The different navigational strategies are depicted under the graphs. C: Circling; ES: Extended Searching; RS: Restricted Searching; LS: Loop Searching; IF: Indirect Finding; F: Finding. (* *p* <0.03; ** *p*< 0.001).

During Place 1 (20 h after injections), latency and peripheral distance values were similar among groups, while

 total distances swum by 7-b96.11 animals were longer than those of 7-sham rats. No differences were observed between 7-b78 and 7-sham groups (Figure 3A-B). Swimming velocities differed significantly among groups, with 7-b78 animals being significantly faster (= 27.82 ± 2.71 cm/sec) than 7-sham rats ( = 22.12 ± 0.57 cm/sec) (*p*<0.03). No significant difference between 7-b96.11 (7-b96.11: = 25.86 ± 1.69 cm/sec) and 7-sham animals was found. The analysis of the navigational strategies revealed that 7-b96.11 rats exhibited the highest percentage of Circling (Figure 3C).

During the Cue phase, 7-b96.11 rats displayed higher latency and total distance values in comparison to 7-sham rats, while no difference was observed between 7-b78 and 7-sham rats (Figure 3A-B). No difference in swimming velocity was found among groups. As for navigational strategies, 7-b96.11 animals still exhibited the highest percentage of Circling (Figure 3C). The high latency and distance values of 7-b96.11 animals may be in part caused by their enhanced Circling. During Place 2, Place 3 and Place 4, latency values, total and peripheral distances and navigational strategies were not statistically different among groups (Figure 3A-B).

In all Probe phases, no difference in the time spent in the previously rewarded quadrant was observed among groups, indicating no mnesic deficits induced by GAD65Ab. One-way and two-way ANOVAs on MWM values are reported (Additional file 2: Table S2 and Additional file 3: Table S3), and relative *post-hoc* comparisons are depicted in Figure 3.

### Animals injected with 5 μg of GAD65Ab

#### Neurological evaluation

Twenty hours after the injections, 5-b96.11 animals exhibited total symptoms more severe than those observed in 5-sham animals. From the second post-injection day onward, no significant differences between groups were detected. No significant difference was found between 5-b78 and 5-sham rats at any time-point. Results with significant differences as established by the non-parametric Mann-Whitney U test on motor assessment are depicted in Additional file 4: Figure S1.

#### Morris Water Maze

No difference among groups was evident for any of the MWM parameters. One-way and two-way ANOVAs on MWM values are reported (Additional file 2: Table S2 and Additional file 3: Table S3).

The main effects of the two epitope-specific GAD65Ab on the various behavioral parameters are summarized in Table 1.

#### Uptake of monoclonal GAD65Ab by AF5 cells

**Figure 4:**
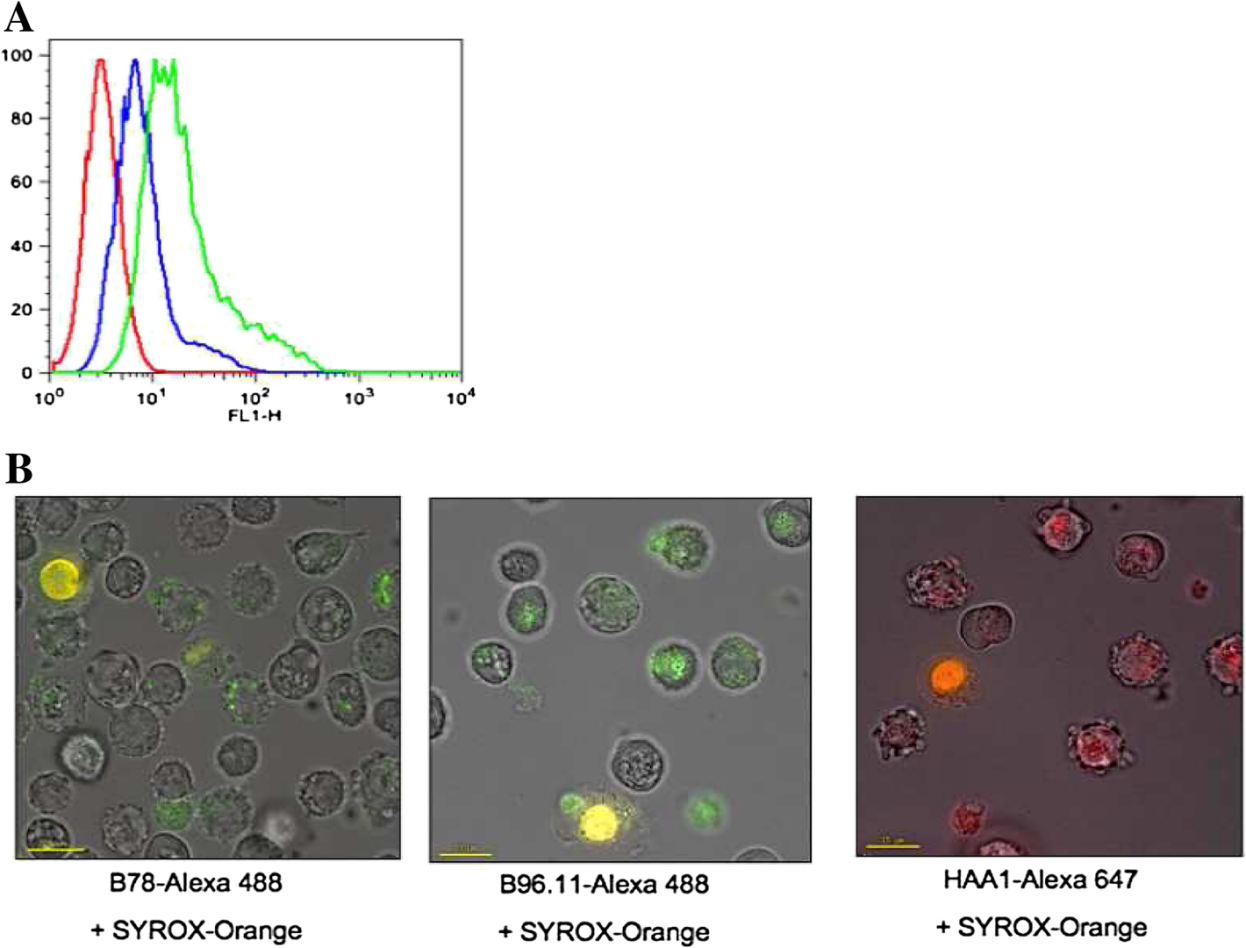
**Uptake of monoclonal GAD65Ab by the rat mesencephalic cell line AF5.** Dose dependent uptake of monoclonal GAD65Ab b78 was assessed by flow cytometry (**A**). AF5 cells were incubated for 4 hours with 10 (blue line) and 50 μg (green line) Alexa-488 b78. Unstained cells are shown in red. Cellular localization of GAD65Ab b78 and b96.11, and monoclonal antibody HAA1-Alexa 647 was assessed by fluorescence and Bright Field microscopy (**B**). AF5 cells were incubated for 4 hours with Alexa-488 b78, Alexa-488 b96.11 or HAA1-Alexa 647 followed by incubation with SYTOX-orange for identification of dead cells. The images reported in the figures are representative of at least three experiments that gave similar results.

Expression of intracellular GAD65 in confluent AF5 cells was confirmed by flow cytometry and immunohistochemistry (data not shown) employing the GAD65-specific monoclonal antibody N-GAD65mAb. This antibody shows no cross-reactivity with GAD67 [29]. Internalization of GAD65Ab by AF5 cells was tested using Alexa-488-labeled b78 and b96.11 and analyzed

 by flow cytometry (Figure 4A). We observed that both antibodies were internalized in a dose-dependent manner. Uptake increased with time, reaching a plateau at 4 hours (data not shown). No uptake was observed at 4°C (data not shown), suggesting that the antibodies were internalized and not surface-bound. This was further confirmed by Fluorescence and Bright Field microscopy, demonstrating intracellular localization of b78 and b96.11 (Figure 4B). Similar findings were observed for human monoclonal antibody HAA1 against Blood group A antigen. Staining with SYTOX-Orange showed that antibodies were taken up by live cells. The cellular distribution of labeled antibodies and SYTOX-Orange clearly shows staining of nuclei with SYTOX-Orange and staining of significantly smaller intracellular structures by the antibodies. Whether these small intracellular structures are in fact vesicles remains to be determined. The mechanism involved in the antibody uptake is currently under investigation.

## Discussion

Intra-cerebellar injections of GAD65Ab affected both motor and cognitive behaviors in an epitope-specific manner. These effects were dose-dependent, given that injections of 5 μg of GAD65Ab induced only mild and transient cerebellar motor symptoms and did not affect MWM performances (Table 1).

Cerebellar dysfunction elicits motor disorders in muscle coordination, balance and muscle strength as well as significant impairment in a variety of cognitive, emotional, and affective functions [32-34]. Previously, we demonstrated that the cerebellum plays a key role in the acquisition of navigational procedural components that control how a new environment is explored [19,35,36]. In MWM, to acquire spatial information hemicerebellectomized rats displayed ineffective explorative strategies, but show unaltered spatial mnesic competencies.

The unilateral (right) intra-cerebellar injections of GAD65Ab evoked postural and procedural effects similar to those induced by a unilateral (right) cerebellar lesion described in clinical [37,38] and experimental reports [19,20,39], although the two monoclonal GAD65Ab induced symptoms different in severity and duration. Namely, b96.11-treated animals showed an initial severe and rapidly decreasing impairment in motor symptoms as well as in navigational strategies (Circling) and b78-treated animals exhibited less severe but long-lasting

**Table 1:** Summary of main effects of epitope-specific GAD65Ab on behavioral parameters measured in this study

	**Neurological evaluation**				**Morris water maze**		
	**Total symptoms**	**Postural symptoms**	**Locomotor asymmetries**	**Complex behaviours**	**Escape latency**	**Total distance**	**Navigational strategies**
**7-b96.11**	Impairment (day1; *p*<0.03)	Impairment (day1; *p*<0.03)	Impairment (day1; *p*<0.03)	Impairment (day1, 3; *p*<0.03)	Increase (Cue; *p*<0.03)	Increase (Place1, Cue; *p*<0.03)	Circling Increase (Place1, Cue; *p*<0.001)
**5-b96.11**	Impairment (day1; *p*<0.03)	No Effect	No Effect	No Effect	No Effect	No Effect	No Effect
**7-b78**	Impairment (day1, 3, 4; *p*<0.03)	No Effect	No Effect	Impairment (day1, 3, 4; *p*<0.03)	No Effect	No Effect	No Effect
**5-b78**	No Effect	No Effect	No Effect	No Effect	No Effect	No Effect	No Effect

 complex motor impairment. Notably, the intra-cerebellar injections of either GAD65Ab did not affect motivational components by modifying the salience of reward (platform) and explorative tendencies. Neither were anxiety levels affected by the GAD65Ab injections, as all groups showed similar levels of peripheral swimming. Recently, it has been described that rats injected multiple times with polyclonal IgG purified from an SPS patient with high GAD65Ab titers exhibited motor symptoms increasing over time [15]. This result fits with the motor deficits we observed in GAD65Ab-treated rats even following a single injection.

To advance a possible mechanism through which GAD65Ab perturbed motor behaviors and navigational competencies, several experimental findings have to be taken into account. In cell and tissue culture systems, IgG from GAD65Ab-positive neurological patients suppressed GABA release from basket cells to Purkinje cells [12,13], changing the balance between glutamate and GABA and possibly causing glutamate excitotoxicity [14,18]. Uptake of antibodies by neurons including Purkinje cells has been recently demonstrated [11] and we confirmed antibody uptake by the AF5 rat mesencephalic cell line. Our recent *in vivo* studies support the cellular uptake of GAD65Ab as b78 injected in the CA3-CA1 area was detected in hippocampal interneurons [40]. Future studies are planned to investigate the cellular localization of monoclonal GAD65Ab injected in the cerebellar nuclei. Previously, we have shown that intra-cerebellar injections of either b78 or b96.11 GAD65Ab increased glutamate cerebellar levels, decreased cerebello-cortical inhibition, and impaired cortico-motor response. Interestingly, these effects were more severe if b78 was injected [18]. Also in the present study b78 appears to trigger longer-lasting effects as compared to b96.11. Based on the observation that only b78 and not b96.11 inhibits the enzyme activity of GAD65, we hypothesize that b78 reduces GABAergic transmission by inhibiting the enzyme activity of GAD65. Alternatively, the GAD65Ab may interfere with the exocytosis of GABA-containing vesicles [14,18]. It is possible that a b96.11-mediated interference with the exocytosis of GABA-containing vesicles will eventually be overcome by increased GABA synthesis, while the longer-lasting effects are the consequence of reduced GABA production mediated by b78. As suggested by *in vitro* experiments, the pathogenic action of GAD65Ab might consist of two phases: an early reversible functional synaptic disorder and a subsequent irreversible excitotoxic degeneration [41,42]. The observation that the effectiveness of immunosuppressant agents on GAD65Ab-positive CA patients depends on the clinical stage or the degree of the disease supports this biphasic action model [43,44]. Whether cerebellar injections with GAD65Ab induce neuronal injury in rats remains to be determined.

A speculative possibility is that GAD65Ab impair the nitric oxide (NO) pathway, which is critical for synaptic plasticity in the cerebellum [45,46] and which is closely related to the activation of NMDA receptors [47,48]. Alterations of biochemical mechanisms underlying cerebellar synaptic plasticity might be involved in the effects reported here [49-51]. A GAD65Ab-mediated alteration of the NO pathway would trigger neurotoxic processes leading to altered behavioral outcome.

## Conclusions

The present findings support the hypothesis that disease-specific GAD65Ab induce distinct levels of impairment in cerebellar circuits leading to motor and procedural symptoms different in severity and duration. Cerebellar nuclei impairment would impact not only the extra-cerebellar targets of cerebello-fugal pathways, but also intrinsic cerebellar nuclear-cortical loops. In conclusion, our study may represent a starting point in the analysis of behavioral effects of epitope specific GAD65Ab in an attempt to characterize neurological syndromes associated with GAD65Ab and aid in the research for more targeted antibody-mediated treatment strategies.

### Availability of supporting data

The data sets supporting the results of this article are included within the article and its additional files.

## Abbreviations

GAD65: 65kDa isoform of Glutamate Decarboxylase; GAD65Ab: GAD65 antibodies; CNS: Central Nervous System; GABA: gamma Aminobutyric acid; NMDA: N-Methyl-Daspartic acid; CA: Cerebellar Ataxia; SPS: Stiff Person Syndrome; T1D: Type 1 Diabetes; MWM: Morris Water Maze; C: circling; ES: extended searching; RS: restricted searching; LS: loop searching; IF: indirect finding; F: finding; PBS: Phosphate Buffered Saline; DMEM: Dulbecco’s Modified Eagle’s Medium; IgG: Immunoglobulin G isotype

## Competing interests

The authors declare that they have no competing interests.

## Authors’ contributions

CSH, LP and MM made substantial contributions to conception, data analysis, interpretation of data and drafted the manuscript. PDB managed the experimental procedures. FF, DC, PC, and DL treated the animals, performed all behavioral evaluations and undertook the statistical analyses. JRR carried out the purification and labeling of GAD65Ab and Flow Cytometry. VV carried out the analysis of internalization of GAD65Ab by Fluorescence and Bright Field microscopy. JH participated in the design of the study and interpretation of data. All authors read and approved the final manuscript.

## Supplementary Material

Additional file 1: Table S1 Behavioral rating scale. **A**. Criteria for evaluation of postural symptoms. **B**. Criteria for evaluation of locomotor asymmetries. **C**. Criteria for evaluation of complex behavior deficits. Click here for file

Additional file 2: Table S2 One-way ANOVAs on MWM values of three experimental groups at 7 or 5 μg dosages. Click here for file

Additional file 3: Table S3 Two-way ANOVAs on MWM navigational strategies of three experimental groups at 7 or 5 μg dosages. Click here for file

Additional file 4: Figure S1 Neurological evaluation of total symptoms (**A**), postural symptoms (**B**), locomotor asymmetries (**C**) and complex behaviors (**D**) in 5-b96.11 (black squares), 5-b78 (grey triangles), and 5-sham (white diamonds) groups. (5-b96.11 *vs.* 5-sham: * *p* <0.03). Click here for file
