# Supplementary material for: Monoclonal antibodies to 65kDa glutamate decarboxylase induce epitope specific effects on motor and cognitive functions in rats
Source: Orphanet J Rare Dis. 2013 Jun 5;8:82. doi: 10.1186/1750-1172-8-82 (PMC3680042; doi:10.1186/1750-1172-8-82)
Supplement: Additional file 1: Table S1 — Behavioral rating scale. A. Criteria for evaluation of postural symptoms. B. Criteria for evaluation of locomotor asymmetries. C. Criteria for evaluation of complex behavior deficits. [file 1750-1172-8-82-S1.doc]

**Table S1.** Behavioral rating scale.

A. Criteria for evaluation of postural symptoms:

| **Score** | **Body tilt** | **Head tilt** | **Nystagmus** | **Limb hyperextension** | **Limb hyperflexion** | **Ankle extrarotation** | **Head bobbing** | **Hypotonia** | **Tremor** |
| --- | --- | --- | --- | --- | --- | --- | --- | --- | --- |
| **0** | Absent | Absent | Absent | Absent | Absent | Absent | Absent | Absent | Absent |
| **1** | Slight | Slight | <20 beats/min | Slight | Slight | Slight | Occasionally present | Slight | Slight |
| **2** | Marked | Marked | <20 beats/min | Marked | Marked | Marked | Repeatedly present | Marked | Marked |

B. Criteria for evaluation of locomotor asymmetries:

| **Score** | **Wide base** | **Collapse**  **on belly** | **Pivoting** | **Circling** | **Steering** | **Side falls** | **Hyperactivity** |
| --- | --- | --- | --- | --- | --- | --- | --- |
| **0** | Absent | Absent | Absent | Absent | Absent | Absent | Absent |
| **1** | Slight tendency | Slight tendency | Rarely present | Occasionally present | Occasionally present | Occasionally present | Slight |
| **2** | Markedly present | Markedly present | Present | Repeatedly present | Repeatedly present | Repeatedly present | Marked |

C. Criteria for evaluation of complex behavior deficits:

| **Score** | **Ascending**  **a ladder** | **Suspension**  **on wire** | **Rearings** | **Vestibular drop** |
| --- | --- | --- | --- | --- |
| **0** | Successful | > 10 sec | Repeatedly present | Present without directionality |
| **1** | Only a few steps | < 10 sec | Occasionally present | Present with side prevalence |
| **2** | Failed | Absent | Absent | Absent |
