# Supplementary material for: Monoclonal antibodies to 65kDa glutamate decarboxylase induce epitope specific effects on motor and cognitive functions in rats
Source: Orphanet J Rare Dis. 2013 Jun 5;8:82. doi: 10.1186/1750-1172-8-82 (PMC3680042; doi:10.1186/1750-1172-8-82)
Supplement: Additional file 2: Table S2 — One-way ANOVAs on MWM values of three experimental groups at 7 or 5 μg dosages. [file 1750-1172-8-82-S2.doc]

**Table S2. One-way ANOVAs on MWM values of three experimental groups at 7 or 5 μg dosages.**

|  | | | **Escape**  **latency** | **Total**  **distance** | **Peripheral**  **distance** | **Mean**  **velocity** | **Time in**  **rewarded quadrant**  **(Probe)** |
| --- | --- | --- | --- | --- | --- | --- | --- |
| **7-b96.11,**  **7-b78,**  **7-sham**  **groups** | Place 1 | df: 2,12 | F= 1.10  *p* n.s. | F= 4.61  *p* =0.03 | F= 1  *p* n.s. | F= 7.47  *p* <0.03 | F= 3.07  *p* n.s. |
|  | **Cue** |  | F= 7.12  *p* <0.03 | F= 12.22  *p* =0.001 | F= 0.18  *p* n.s. | F= 2.82  *p* n.s. |  |
|  | **Place 2** |  | F= 1.30  *p* n.s. | F= 1.31  *p* n.s. | F= 2.95  *p* n.s. | F= 0.65  *p* n.s. | F= 3.22  *p* n.s. |
|  | **Place 3** |  | F= 1.45  *p* n.s. | F= 1.29  *p* n.s. | F= 1.53  *p* n.s. | F= 0.41  *p* n.s. | F= 1.64  *p* n.s. |
|  | **Place 4** |  | F= 0.82  *p* n.s. | F= 1.66  *p* n.s. | F= 0.23  *p* n.s. | F= 0.41  *p* n.s. | F= 0.07  *p* n.s. |
| **5-b96.11,**  **5-b78,**  **5-sham**  **groups** | Place 1 | df: 2,12 | F= 2.50  *p* n.s. | F= 1.22  *p* n.s. | F= 0.83  *p* n.s. | F= 1.09  *p* n.s. | F= 1.21  *p* n.s. |
|  | **Cue** |  | F= 3.15  *p* n.s. | F= 1.87  *p* n.s. | F= 0.74  *p* n.s. | F= 2.64  *p* n.s. |  |
|  | **Place 2** |  | F= 1.11  *p* n.s. | F= 0.67  *p* n.s. | F= 1  *p* n.s. | F= 1.64  *p* n.s. | F= 0.52  *p* n.s. |
|  | **Place 3** |  | F= 1.03  *p* n.s. | F= 0.63  *p* n.s. | F= 0.21  *p* n.s. | F= 0.58  *p* n.s. | F= 0.37  *p* n.s. |
|  | **Place 4** |  | F= 3.27  *p* n.s. | F= 2.77  *p* n.s. | F= 0.49  *p* n.s. | F= 0.31  *p* n.s. | F= 0.31  *p* n.s. |
