# Supplementary material for: Monoclonal antibodies to 65kDa glutamate decarboxylase induce epitope specific effects on motor and cognitive functions in rats
Source: Orphanet J Rare Dis. 2013 Jun 5;8:82. doi: 10.1186/1750-1172-8-82 (PMC3680042; doi:10.1186/1750-1172-8-82)
Supplement: Additional file 3: Table S3 — Two-way ANOVAs on MWM navigational strategies of three experimental groups at 7 or 5 μg dosages. [file 1750-1172-8-82-S3.doc]

**Table S3. Two-way ANOVAs on MWM navigational strategies of three experimental groups at 7 or 5 μg dosages.**

|  |  | | **Group Effect**  (df: 2, 12) | | **Strategies**  **Effect**  (df: 5, 60) | **Group x**  **Strategies**  (df: 10, 60) |
| --- | --- | --- | --- | --- | --- | --- |
| **7-b96.11,**  **7-b78,**  **7-sham**  **groups** | | Place 1 | | F= 2.15  *p* n.s. | F= 4.91  *p* <0.001 | F= 5.79  *p* <0.00001 |
|  |  | **Cue** | | F= 3.76  *p* n.s. | F= 3.19  *p* <0.03 | F= 5.13  *p* <0.0001 |
|  |  | **Place 2** | | F= 3.16  *p* n.s. | F= 4.88  *p* <0.001 | F= 1.54  *p* n.s. |
|  |  | **Place 3** | | F= 0.66  *p* n.s. | F= 6.96  *p* <0.0001 | F= 1.51  *p* n.s. |
|  |  | **Place 4** | | F= 1.04  *p* n.s. | F= 16.10  *p* <0.00001 | F= 0.35  *p* n.s. |
| **5-b96.11,**  **5-b78,**  **5-sham**  **groups** | | Place 1 | | F= 0.35  *p* n.s. | F= 21.40  *p* <0.00001 | F= 0.50  *p* n.s. |
|  |  | **Cue** | | F= 0.57  *p* n.s. | F= 6.26  *p* <0.0001 | F= 1.09  *p* n.s. |
|  |  | **Place 2** | | F= 1.60  *p* n.s. | F= 4.78  *p* <0.01 | F= 0.76  *p* n.s. |
|  |  | **Place 3** | | F= 0.15  *p* n.s. | F= 17.60  *p* <0.00001 | F= 0.58  *p* n.s. |
|  |  | **Place 4** | | F= 3.14  *p* n.s. | F= 28.13  *p* <0.0001 | F= 0.89  *p* n.s. |
