# Supplementary figures and images for: Monoclonal antibodies to 65kDa glutamate decarboxylase induce epitope specific effects on motor and cognitive functions in rats
Source: Orphanet J Rare Dis. 2013 Jun 5;8:82. doi: 10.1186/1750-1172-8-82 (PMC3680042; doi:10.1186/1750-1172-8-82)

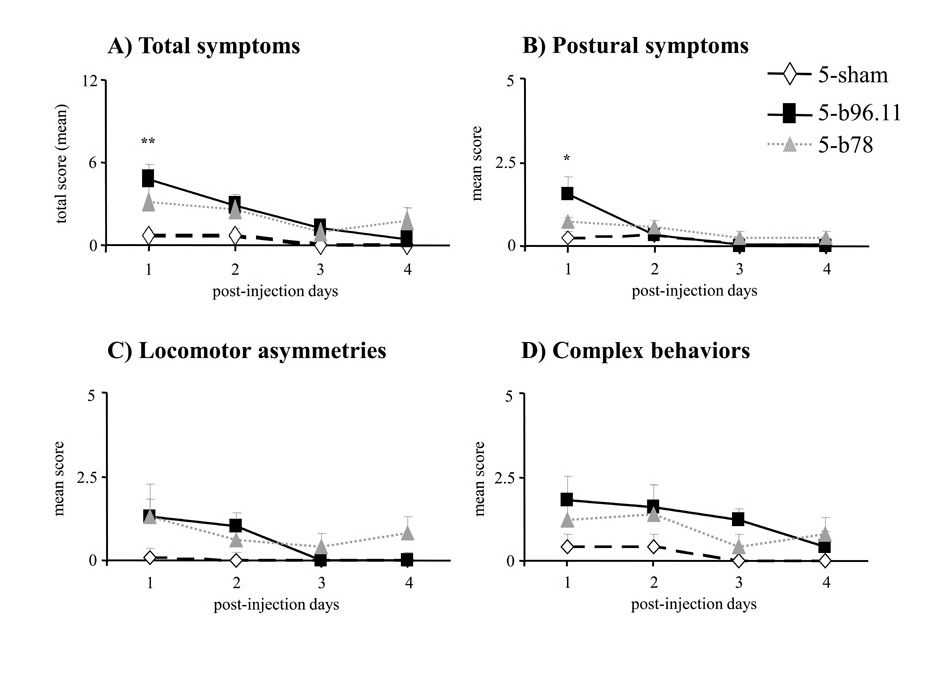

Supplement: Additional file 4: Figure S1 — Neurological evaluation of total symptoms (A), postural symptoms (B), locomotor asymmetries (C) and complex behaviors (D) in 5-b96.11 (black squares), 5-b78 (grey triangles), and 5-sham (white diamonds) groups. (5-b96.11 vs. 5-sham: * p <0.03). [file 1750-1172-8-82-S4.jpeg]
